# The Minimum 5-Year Follow up of a Highly Versatile Distally Anchored Femoral Revision System With Hydroxyapatite Coating

**DOI:** 10.1016/j.artd.2023.101185

**Published:** 2024-01-26

**Authors:** James Shelton, ARCOS Study Collaborative, Jonathon Barrow, Keijo Mäkelä, Petri Virolainen, Vijay Killampalli, Francesco Angles- Crespo, Urban Hedlundh, Marti Bernau, Jari Mokka, Andrew Gordon

**Affiliations:** Sheffield Teaching Hospitals NHS Foundation Trust, Yorkshire, UK

**Keywords:** Revision, Femur, Hip, Infection

## Abstract

**Background:**

Total hip arthroplasty is one of the most successful operations medicine can offer. As more patients undergo total hip arthroplasty the revision burden increases proportionately. This is a cohort study of the Arcos Modular Femoral Revision System.

**Methods:**

The primary outcome was rerevision of the femoral component. Secondary outcomes include complications and radiological and clinical outcomes over 5 years.

**Results:**

A total of 74 patients were recruited, and the femoral survival rate was 100% at 5 years with 12 censorships. A total of 31 patients were given Proprosky 3/4 preoperatively. Eleven patients underwent further procedures; however, no femoral components were revised. Kaplan-Meier analysis was performed by a biostatistician. Patients demonstrated a consistent and sustained improvement in Harris hip score, Oxford hip score, and EQ-5D. Radiological review revealed minimal and stable lysis around the femoral components.

**Conculsion:**

The ARCOS Modular Femoral Revision System was designed to offer a range of options to allow femoral bone stock preservation and avoiding conversion to an endoprosthetic and seems to be effective in the medium term.

## Introduction

As medical care advances so does the long-term survival of patients receiving primary arthroplasty. There is also an increasing evidence base for arthroplasty in young adults and even children [[1], [2], [3]]. These successes in patient management also leads to an increasing burden of revision surgery [4]. The increasing complexity of revision arthroplasty has seen a move from monoblock revision stems to highly modular platforms [5]. Flexibility in femoral revision systems has become a key discriminator between different brands. Occasionally these revision platforms can be useful in complex primary arthroplasty as well.

The Zimmer Biomet ARCOS Modular Femoral Revision System has been specifically designed with a wide range of modular options to manage the majority of femoral revision scenarios up to endoprosthetics. Whilst the existing revision platforms demonstrate good medium-term survivorship (89.3%-94% survivorship at 10 years) [6,7], other systems offered parity or improvement in terms of survivorship and flexibility [[8], [9], [10]]. There are limited data on femoral bone stock preservation, and it is reasonable to assume that in some salvage situations the proximal femur was deemed nonviable and an endoprosthesis was used. These cases would subsequently be excluded from the published literature on revision stem survivorship.

Introduced in 2010 the ARCOS system comprises 5 stem designs and 3 body designs in an array of lengths and sizes resulting in 117 combinations to treat a wide variety of pathologies required for femoral reconstruction. Stem designs include splined tapered, slotted, bullet tipped, interlocking, and extended trochanteric osteotomy (ETO) variants and body designs include coned, calcar bearing, and broached designs. The splined tapered stem is grit blasted and the other 4 stems and all proximal bodies all feature the Zimmer plasma porous spray coating to provide initial “scratch fit” stability pending bony on growth. In this study, all stems and proximal bodies were coated in BoneMaster hydroxyapatite.

In this study, we present the minimum 5-year survivorship of the ARCOS system for revision arthroplasty.

## Material and methods

The study was designed as a prospective, multicenter, multinational, longitudinal cohort study. Centers were identified in the United Kingdom, Spain, Sweden, and Finland. Data were collected as preoperative and perioperative findings, clinical, radiological, and survival data. Due to difficulty in recruitment, 38 patients were recruited retrospectively documenting the preoperative and perioperative findings from the case notes. The remaining 36 patients were recruited prospectively. A study protocol (Appendix 1) was designed, and the appropriate research and ethics permissions gained from all recruitment sites (IRAS UK, TURKIJA Finland, SNCME Sweden, and CEIC Spain). The primary investigator for all sites was required to obtain an independent ethics committee review of the project prior to initiation at each site and provide a copy of the written approval to the sponsor.

The primary outcome of this study was to determine the 5-year survival of the ARCOS modular femoral revision system for all-cause revision/complex primary total hip arthroplasty.

The secondary outcome measures of this study include complication profile and radiographic and clinical outcomes of patients hip revision surgery using the ARCOS system. Provisions were made in the protocol to allow implant retrieval for failures however this was ultimately not required.

The sample size was determined by the projected sales target from Zimmer Biomet over a 5-year period. A nominal target of 100 cases with a 5-year follow up was set based upon these numbers.

Our patient population comprised all-cause femoral revision in which an ARCOS stem was used during the recruitment phase. There was also one patient who had an ARCOS stem for an atypical femur secondary to hip dysplasia. Whilst not a specified exclusion criterion, no patients underwent a single stage revision for infection.

An information booklet was provided along with a verbal explanation of the study to ensure informed consent and a consent form signed by all participants and kept by Zimmer Biomet. The voluntary nature of the study was communicated, and consent could be rescinded at any time during the study.

Data were collected from study participants at set time points along the follow-up pathway as described below:

Preoperative patient demographics, reason for revision, and hip scores were collected. The patients’ radiographs were assessed for lucency around the acetabular component (zones 1-3 Charnley and Delee), lucency around the femoral component (zones 1-14 Barrack), and subsequently classified using the Paprosky classification of bone loss in the femur.

Intraoperative data were gathered on surgical approach, components used on femoral, acetabular, and bearing surfaces of the joint and intraoperative complications. The surgical technique was adapted from the Zimmer Biomet operation technique PDF and reaming (power vs manual) was surgeon choice. Both techniques are represented within the study population.

Postoperatively, patients were invited to follow up at intervals of 6 weeks, 1 year, 3 years, and 5 years. At each assessment patients were evaluated clinically, with Harris Hip Score, Oxford Hip Score, and EQ5D patient-reported outcome measures. Postoperative complications both acute and delayed were reported. Patients were radiographically assessed by the base hospital engaged in the study for lucency around the cup and the stem for indications of failure. Survivorship of the implant was assessed using a Kaplan-Meier survival analysis by a professional biostatistician.

The baseline, operative, surgical, and clinical outcome data were summarized by descriptive statistics based on the data scale. The categorical data were summarized by frequency and percentage. The continuous data were analyzed by mean and standard deviation along with samples size N. Kaplan-Meier survival analysis was conducted to provide the survival estimates up to 6 years after surgery with event definition as revision. As safety evaluation, the detail listing of device-related intraoperative and postoperative complications and all other non-device related complications were provided in the report. The software used for the analysis was SAS Enterprise Guide, version 7.13HF8, copyright by SAS Institute Inc., NC., USA.

## Results

**Figure 1:**
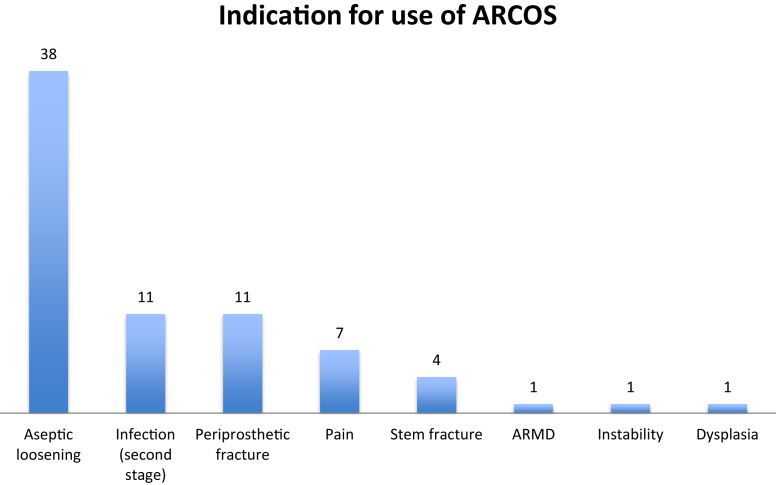
Indication for use of ARCOS femoral revision system.

A total of 38 patients were recruited retrospectively and 36 patients prospectively. Across all study sites, a cohort of 74 patients were recruited across 5 study sites (2 in the United Kingdom, 1 in Finland, 1 in Spain, and 1 in Sweden). Patient demographics demonstrated a mean age of 69.7 ± 11.5 (30-87) years, mean body mass index of 29.2 ± 6.4 (19.3-59.3) kg/m^2^, and a female-to-male ratio of 1.05:1.0 (F38:36M). The mean follow-up across all patients was 4.2 ± 1.6 (0.1-6.6) years. Indications for revision of femoral component are demonstrated in Figure 1 below.

**Figure 2:**
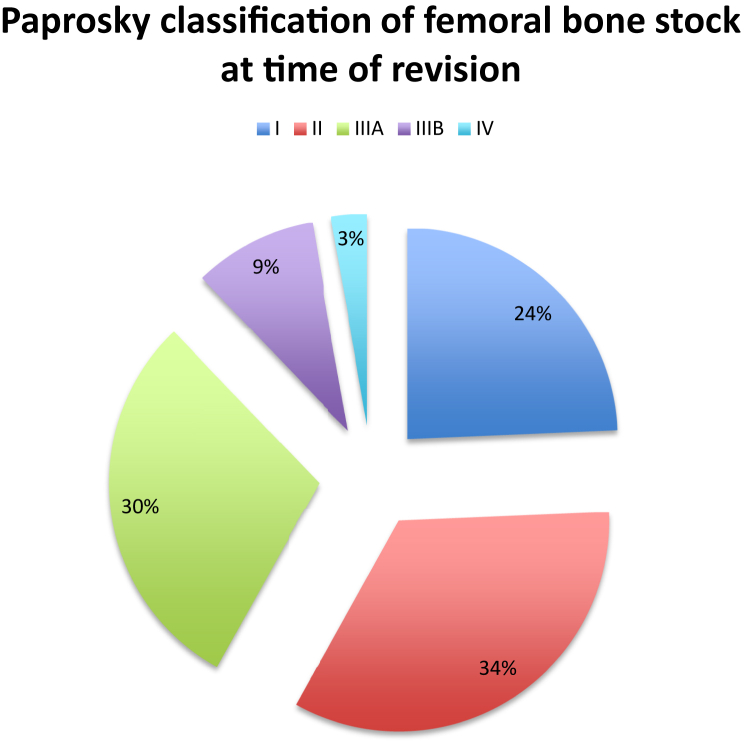
Paprosky classification of femoral bone stock at time of revision.

Femoral bone stock at the time of revision is detailed in Figure 2.

Acetabular lucency was noted in 16 patients demonstrating a mean of 2 mm ± 3 mm (1 standard deviation) in zone 1, 1.1 mm ± 2.2 mm in zone 2, and 1.5 mm ± 3.3 mm in zone 3.

Preoperative clinical evaluation revealed a mean Harris hip score of 41.4 ± 15.3(1 standard deviation), an EQ5D of 0.5 ± 0.2, and an Oxford hip score of 18.8 ± 8.6.

Perioperative findings demonstrated the majority of patients (46) underwent revision through a posterior approach, a further 20 patients had a posterior approach along with an ETO and finally 8 patients were revised through an anterolateral approach.

In all the cases of the study, cup and stem were revised.

Components used during the revision included 70 splined tapered stems, 3 interlocking stems, and 1 extended trochanteric osteotomy stem. All patients received a cone type proximal body. A wide array of acetabular components were used; 68 were revision shells, 2 were dual mobility monoblock shells, and 4 were triflanged or custom components. Bearings were either metal on polyethylene (28), ceramic on polyethylene (14), or not documented (32).

There were 46 hip-related complications recorded in 36 patients which are summarized in Table 1; of these, 10 could be considered “device and instrumentation related” (trochanteric and femoral fractures, metalwork irritation, trochanteric, thigh and hip pain). An additional 85 healthcare contacts were noted for nonimplant problems, for example, abdominal pain, cardiac symptoms, cerebrovascular events, and so on. Interval patient reported outcome measures demonstrated a sustained improvement in OHS, HHS and EQ5D across all time points (Table 2).

**Table 1:** Hip-related complications in study patients.

Intraoperative complications		Immediate postoperative complications		Postoperative complications	
Sciatic nerve palsy	1	Persistent wound drainage	5	Second operation	11
Skin burn from cement removal	1	Bruising/haematoma	3	Dislocation	6
Femoral fracture	1	Wound dehiscence	1	Deep infection necessitating debridement and implant retention	4
Trochanteric fracture	1	Dressing allergy	1	Metalwork irritation	3
Vascular injury	1			Trochanteric pain	3
				Iliac crest pain	2
				Thigh pain	1
				Hip pain	1

**Table 2:** Patient-reported outcome measures preoperatively and throughout study.

Outcome	Average (SD)				
	Preoperative	6 wk postoperation	1 y postoperation	3 y postoperation	5 y postoperation
Harris hip score	41.3 (15.7)	60.0 (15.3)	73.1 (21.3)	78.1 (18.9)	79.5 (16.5)
EQ5D	0.5 (0.2)	0.7 (0.2)	0.8 (0.2)	0.8 (0.2)	0.8 (0.2)
Oxford hip score	18.8 (8.6)	28.3 (8.8)	34.4 (10.9)	36.9 (10.5)	39.2 (8.7)

**Figure 3:**
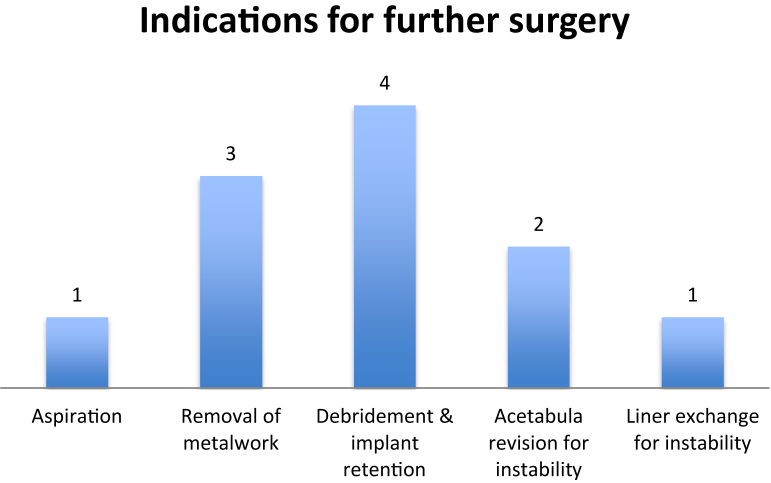
Indications for further surgery.

Further surgeries are demonstrated in Figure 3.

The following 3 removals of metalwork were noted: 1 migrated cerclage wire, 1 screw with soft-tissue irritation, and 1 loose trochanteric reattachment bolt. It is also worth noting that another trochanteric reattachment bolt was noted to be loose and free in the soft tissues, which was managed expectantly. A total of 28 trochanteric reattachment plate/bolts were used during the study with 2 noted failures (7%).

Radiographic analysis demonstrated a maximum of 0.4 mm lysis at 5 years in any Barrack zones of the femoral component with 6 zones 0 mm, 2 zones 0.1 mm, 4 zones 0.2 mm, 1 zone 0.3 mm, and 1 zone 0.4 mm. No subsidence was noted by any study sites.

**Figure 4:**
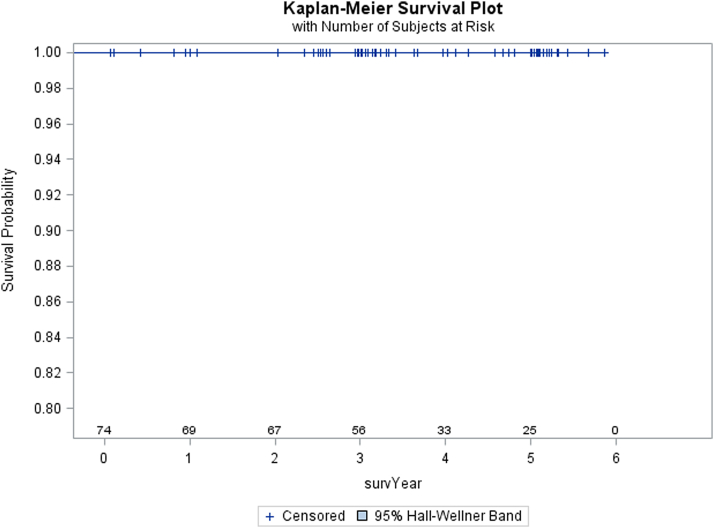
Kaplan Meier survival chart for the Arcos femoral system.

The revision rate at 5 years was 0 cases. Over the course of the study, 4 patients rescinded consent for the study and 8 patients died leading to a total of 12 patients being censored over the course of the study from the Kaplan-Meier analysis demonstrated in Figure 4.

## Discussion

This study demonstrates excellent mid-term survival of the ARCOS modular femoral revision system comparative to the other options on the market such as the ZMR (93.5% at 5 years and 89% at 10 years), SROM (60%-95% at 10 years), Wagner SL (92% at 10 years), MRP Titan (97% at 10 years), and the Anatomic Medullary Locking (95% at 10 years). The vast majority of cases in this study used the splined tapered stem. Additional flexibility in revision cases can result in preservation of femoral bone stock which would otherwise require endoprosthetic replacement. The survivorship of the stem despite the high numbers of Proprosky 3 and 4 femurs (n = 31) indicated the capability of the system to bridge the gap between traditional diaphyseal bearing revision stems and endoprosthetics potentially allowing preservation of bone stock and more options in the future. The distal interlocking stem in particular allows for use in uncertain diaphyseal grip to provide initial stability pending full integration of the stem through bony on-growth. The complication profile of the cases is consistent with the complex nature of revision surgery [11]. The patients requiring an ARCOS stem included 4 patients with confirmed postoperative infection <6 weeks postoperatively (mean 34 days) necessitating a debridement and implant retention procedure. These procedures being successful seem to indicate rapid integration of the femoral component as it is not possible to postoperatively debride the stem.

The improvement in clinical and radiological outcome measures are sustained across the studies duration indicating well-integrated components. The 5-year survival of the ARCOS is superior to other revision stems with the porous plasma coatings [12,13]. The majority of failures reported by McInnes in 2020 demonstrate stem subsidence (failure of integration) or aseptic loosening in both porous coated cylindrical stems at a mean time of 4.2 years and titanium tapered stems at a mean time of 4.4 years [6]. Van Houwelingen in 2012 [14] also described failures in modular fluted stems at 7 years although interestingly these were predominantly junctional stem fractures. The lack of subsidence, stem fractures, or taper failures at this time point is encouraging for long-term survival. Further observation of this cohort is required to assess longer-term survival especially in the type IIIB and IV where distally fixed but unsupported modular junctions could fail in time.

## Conclusions

This study demonstrates excellent survivorship at 5 years of the ARCOS femoral revision system for both aseptic and 2-stage septic revisions with significant bone loss. Further analysis is required to follow up this cohort long term to ensure that the survival advantage is maintained.

### Limitations

This study failed to reach the initial recruitment target of 100 patients gaining a cohort of 74 patients. Use of multiple international sites also has led to discrepancies in the data due to differing nomenclature between sites, these have been categorized by the authors to provide ease of data translation.

This study whilst intending to use a spectrum of the ARCOS system only pertains to the splined taper stem (n = 70), the interlocking stem (n = 3), the ETO stem (n = 1), and the cone type proximal body. The other 2 body and stem types were not assessed in this project as a result of surgeon choice at the time of revision. Whilst produced on the same technology platform and possible to extrapolate outcome results from this, we must be clear that this study only contains 3 stem types and 1 body type. Even with this spread of implants, we must be cautious of the results in low number stem types (interlocking and ETO).

## Appendix A Supplementary data

Conflict of Interest Statement for Shelton

Conflict of Interest Statement for Gordon
