# Supplementary material for: The Minimum 5-Year Follow up of a Highly Versatile Distally Anchored Femoral Revision System With Hydroxyapatite Coating
Source: Arthroplast Today. 2024 Jan 26;26:101185. doi: 10.1016/j.artd.2023.101185 (PMC11239970; doi:10.1016/j.artd.2023.101185)
Supplement: Appendix 1 [file mmc3.pdf]

Arcos® Revision Stem: Evaluation of Clinical Performance

PROTOCOL NUMBER (Study ID): GBMET.CR.US19.12

PROTOCOL VERSION: v. 6 (March 18, 2015)

GENERAL INFORMATION

Study Sponsor(s): BIOMET

Sponsor Contact information can be found in the investigator binder.

A list of name and title of the investigator(s) who is (are) responsible for conducting the study, and the address and telephone number(s) of the investigational site(s) will be maintained by each participating Biomet SBU or subsidiary.

Table of Contents

1. INTRODUCTION

1.1. BACKGROUND

1.2. DEVICE DESIGN AND DESCRIPTION*

1.3. RATIONALE FOR CURRENT STUDY

1.4. STUDY PURPOSES

2. STUDY DESIGN

2.1. OVERALL DESIGN

2.2. STUDY GROUPS/TREAMENTS

2.3. NUMBER OF SITES AND SUBJECTS/PROCEDURES

2.4. EFFICACY AND/OR SAFETY HYPOTHESES

2.5. PRIMARY AND SECONDARY ENDPOINTS

2.6. ASSESSMENT PROCEDURE

2.6.1. ASSESSMENT PARAMETERS AND METHODS

2.6.2. ASSESSMENT TIMELINES/SCHEDULE

2.6.3. ALLOWED WINDOW OF EACH SCHEDULE

2.7. STUDY DURATION

3. SELECTION AND WITHDRAWAL OF SUBJECTS

3.1. INCLUSION CRITERIA

3.2. EXCLUSION CRITERIA

3.3. SUBJECT WITHDRAWAL

4. PROTOCOL DEVIATION MANAGEMENT AND REPORTING

5. ADVERSE EVENT MANAGEMENT AND REPORTING

6. IMPLANT RETRIEVAL AND ANALYSIS OF REMOVED IMPLANTS

7. SAMPLE SIZE JUSTIFICATION

8. CONTROL OF INVESTIGATIONAL DEVICE

9. DATA COLLECTION, HANDLING AND RETENTION

9.1.SOURCE DOCUMENT REQUIREMENTS

9.2.CASE REPORT FORMS

9.3. ELECTRONIC DATA ENTRY

9.4 STUDY DOCUMENT RETENTION

10. DATA REPORTING

11. RISK ANALYSIS

12. MONITORING PLAN

12.1 FREQUENCY

12.2 SAMPLING PLAN

12.3 MONITORING TASKS

12.4 STUDY CLOSE-OUT

13. LABELING

14. ETHICAL AND REGULATORY REQUIREMENTS*

14.1. CODE OF CONDUCT

14.2. REGULATORY APPROVAL (i.e. FDA, MHRA, MHLW/PMDA*)*

14.3. INSTITUTIONAL REVIEW BOARDS

14.4. INFORMED CONSENT

14.5. SUBJECT CONFIDENTIALITY

15. INSURANCE AND INDEMNIFICATION

16. STUDY DEFINITIONS

17. REFERENCES

18. APPENDICS

**THE ORDER OF SECTIONS MAY BE RE-ARRANGED PER APPLICABLE REGULATIONS*

STUDY SUMMARY

TITLE Arcos Revision Stem: Evaluation of Clinical Performance

DESIGN Retrospective and Prospective Multi-Center Two Armed Non-Comparative Trial

PURPOSE The primary purpose of this study is to evaluate the clinical performance of the Arcos Revision Stem system, determine the stability of the implants, and evaluate any relationship between bone defect level and the success of the Arcos Stem.

OUTCOME MEASURES Primary Endpoint: survivorship and revision rate up to 5 years

Secondary Endpoints: Stability and Fixation of Arcos Hip by radiographic assessment , Relationship between bone defect level, Oxford hip score and Harris hip score postoperative,
Adverse events, complications.

Case Report Forms: Demographic Data, Paprosky Acetabular/Femoral Defects, Operative Data, Oxford Hip Score, Harris Hip Score, Collection of X-rays, Radiographic Evaluation form, Adverse Events, Revisions, Lost to Follow-up, and Protocol Deviations

POPULATION 200 Patients: 100 with BoneMaster HA, 100 without BoneMaster HA

Up to ten sites will be used to fulfill enrollment to this study

All cases enrolled will be those implanted with either the Cone, Broached, or Calcar Proximal Body and one of the five distal options (Slotted, Bullet-tip, Interlocking, STS, or ETO).

ELIGIBILITY To be included in the study, a patient have received a revision total hip arthroplasty with the Arcos system. The Arcos system is to be used in accordance to the indications for use and contraindications detailed in the approved labeling of the device.

DURATION 5 years follow up

Table of Contents

1. INTRODUCTION

1.1. BACKGROUND

Revisions involving the femur continue to be one of the most difficult operations faced by surgeons today. While the published clinical results for revision total hip arthroplasty have shown favorable results, leg length discrepancies and instability are still a concern. Leg length discrepancies and instability can be addressed by implant design. One-piece and modular femoral stems have been used for the past twenty years to address femoral revision cases. However, modular implants provide surgeons the flexibility to adjust leg length and apply the necessary anteversion or retroversion to address stability. In addition, both metaphyseal and diaphyseal defects may be addressed independently.

Biomet was at the forefront of implant design for revision hip arthroplasty with the introduction of the Modular Calcar System in 1992. This revision system was one of the first modular systems to address complex femoral reconstruction with a porous coated, modular calcar-replacing proximal body and modular distal stem segments. Biomet was also the first company to address taper strength at the modular junction with the introduction of Roller Hardening Technology in 2000. The Arcos® Modular Femoral Revision System builds upon the clinical success of this platform, design philosophies and technology while expanding the options available to address varying needs of patients and surgeons.

1.2. DEVICE DESIGN AND DESCRIPTION

The Biomet® Modular Revision Femoral System (Arcos Hip) is a comprehensive, press-fit revision stem design that provides the surgeon with multiple styles of modular proximal and distal bodies for reconstruction of various defects commonly seen in femoral revision surgery.

The proximal bodies will consist of broached, calcar-replacing, and cone-style implants. All proximal bodies, made from Ti-6Al-4V (ASTM F-136 or F-620), will include features for insertion and extraction, as well as a female taper for attaching to the distal stems. The proximal implants will be offered in a range of sizes with standard and high offset neck geometries.

The system offers five distal geometry options to address differing quality of diaphyseal bone, desired fixation and surgical technique. These are:

- Slotted
- Bullet-tip
- Interlocking
- STS (Splined Tapered Stem)
- ETO (Extended Trochanteric Osteotomy)

The distal stem, made from Ti-6Al-4V (ASTM F-136), will include either a porous coating or grit blast surface. The distal stems will be offered in a range of diameters from 12-30mm and a range of lengths including 115-300mm, depending on the style.

All proximal bodies and distal stems are available without Bonemaster coating for use in the U.S. and with Bonemaster hydroxyapatite coating for use in the E.U,

The system also includes auxiliary implants to aid in fixation: trochanter reattachment claws, bolts, and interlocking screws. The system also features a single set of instrumentation for all styles. The system is intended for uncemented applications.

Overall length of proximal and distal assemblies range from 165mm to 380mm, depending on chosen combination of implants.

1.3. RATIONALE FOR CURRENT STUDY

This study intends to evaluate the safety and performance of Arcos Hip system in terms of function improvement postoperatively, stability and fixation of femoral stems and survivorship.

This study will be conducted in compliance with this protocol, applicable corporate procedures, internal SOP and applicable regulations.

1.4. STUDY PURPOSES

The primary objectives of the study are to:

- Evaluate clinical performance of various options of Arcos Hip Modular Femoral Revision System (“Arcos hip”) in hip revisions
- Determine the stability and fixation of Arcos hip implants.

- Assess any relationship between bone defect level, effectiveness of treatment and usage of various options of Arcos hip.
- Collect survivorship data

2. STUDY DESIGN

2.1. OVERALL DESIGN

The study will consist of patients implanted with the Cone, Broached, or Calcar Proximal Body with one of the five Distal options. The Distal options include the Slotted, Bullet-tip, Interlocking, STS, and ETO Distal Stems. Patient demographic, preoperative clinical outcomes and operative information will be collected retrospectively. Postoperative clinical outcome, radiographic assessment and adverse events will be collected either retrospectively or prospectively up to 5 years follow up, depending on the individual length of follow-up from each patient. A total of 200 cases from up to 10 sites in the U.S., EU, CAN, & AUS will be enrolled and followed. Enrollment will end when the maximum enrollment of 200 cases has been met.

2.2. STUDY GROUPS/TREAMENTS

The study will consist of two patient groups. One group will contain 100 cases using BoneMaster HA coating from the EU and the other group will consist of 100 cases without BoneMaster HA from the U.S. All cases enrolled will be those implanted with a Broached, Cone, or Calcar Proximal Body with one of the five distal options.

2.3. NUMBER OF SITES AND SUBJECTS/PROCEDURES

The sample size is 100 hips from each group. Up to 10 sites will be used for enrollment.

Arcos Hip without BM HA	***100***
Arcos Hip with BM HA	***100***

2.4. EFFICACY AND/OR SAFETY HYPOTHESES

Not applicable as this is a Post Market Study

2.5. PRIMARY AND SECONDARY ENDPOINTS

Primary Endpoints:

Survivorship and revision rate up to 5 years

Secondary Endpoints:

- Postoperative restoration of femoral offset and leg length
- Radiographic measurements of stability and fixation, outcome, incidence of radiolucencies around the prosthesis and bone remodeling
- Relationship between bone defect level and survivorship
- Harris Hip Score, Oxford Hip Score
- Complications

2.6. ASSESSMENT PROCEDURE

Retrospective collection of

A. Preoperative information
    a. Historical Record and demographic data include but are not limited to gender, age at surgery, height, weight, primary diagnosis
    b. Paprosky Classification of Femoral Defect
    c. Papsroky Classification of Acetabular Defect
B. Operative information
    a. Operative Record includes but are not restricted to date of surgery, surgical approach, implant components (part & lot number)
C. Immediate Post-Op information
    a. Complications
    b. Radiographic assessment (full pelvic AP and med. lateral view)
D. Discharge:
    a. Discharge Form (EU only)
E. Retrospective/prospective collection of 6-12 months, 2-3 years and 5 years follow up
    a. Harris Hip Score
    b. Oxford Hip Score
    c. Radiographic Assessment – Full Pelvis and lateral

2.6.1. ASSESSMENT PARAMETERS AND METHODS

A. Preoperative Data:

1. *Enrollment Form*: A form detailing the inclusion and exclusion criteria will be used to screen patients for participation into the study. Since the study is de-identified, this is also a compliance report for the site to report to the Sponsor that the patients has been screened for compliance and also signed a copy of the Informed Consent, and it is on file at the study site.

2. *Informed Consent*: Each patient must sign an Informed Consent that is approved by the appropriate authority as required by the national regulations of the country in which the evaluation is taking place (e.g: IRB, Ethical Committee, etc).

3. *Historical Record*: Demographic, diagnostic and medical history information will be collected on each patient. However patient identifiers that could be considered “PHI” (Patient Health Information) will not be reported to the study sponsor, Biomet.

B. Clinical Assessments

1. *Operative Record*: An operative record will be completed to record details of each operative procedure and of the used implants.

2. *Outcomes Assessments*: Data to determine the clinical and functional performance of the device will be collected. (Harris Hip Score, Oxford Hip Score).

C. Radiographic Assessments (including Radiographic Assessment protocol)

1. Views:
    a. Full Pelvic Anteroposterior (AP)
    b. Lateral
2. Placement:
    a. Full Pelvic Anteroposterior
        i. This radiographic view will be obtained with the patient placed in the supine position with the bilateral hip joint in a neutral position. The radiation beam must be centered on the pubic symphysis. The X-ray

should include the total prosthesis, including the entire length of the femoral stem.

b. Lateral
   i. A cross-table or frog-leg lateral radiograph will be obtained with the patient placed in the supine position with the contralateral hip flexed to 90° or maximally. The direction of the radiation beam is parallel to the examination table and is at 45° to the long axis of the body. Again, the X-ray should include the total prosthesis, including the entire length of the femoral stem.

3. Copies:
   a. Copies should be de-identified of all patient health information and labeled with the Joint Assist Patient ID Number.
   b. Can be sent to Biomet in any of these acceptable fashions:
      i. Actual copies mailed
      ii. Digital copies saved to disc and mailed (dicom format)
      iii. Instant transfer of images to Biomet's secure FTP server.

2.6.2. ASSESSMENT TIMELINES/SCHEDULE (*tabulated format preferred*)

	**Pre-Op**	**Intra-Op**	**Immediate Post-OP**	**6-12 month 2-3 year 5 year Follow-Up**
Historical Record	X			
Paprosky Defect Forms	X			
Operative form		X		
Harris Hip Score				X
Oxford Hip Score				X
Radiographic assessment			X	X

Complications, LTF, & Protocol Deviations	Anytime

2.6.3. ALLOWED WINDOW OF EACH SCHEDULE (*tabulated format preferred*)

Follow-up Time Point	Allowed Window
6 -12 months	5 months - 18 months
2-3 Year	19 months - 54 months
5 Year	> 55 months

Each follow-up visit time point will be determined based on the date of surgery

2.7. STUDY DURATION

All 200 Arcos Modular Femoral Revision cases will be followed up to 5 years. The study is expected to last for 5 years.

3. SELECTION AND WITHDRAWAL OF SUBJECTS

Inclusion/exclusion criteria are identical to those indications and contraindications stated in the package insert of Arcos hip Implants

3.1. INCLUSION CRITERIA

Patients will be included in this study if they received the Arcos Modular Femoral Revision System per the approved indications for use for Arcos specifically.

- Patients undergoing Revision of previously failed total hip arthroplasty.

The Arcos™ Modular Femoral Revision System hip components are single-use implants, intended for uncemented use only.

Only subjects who have received the Arcos Revision Stem System will be included in this outcomes study.

3.2. EXCLUSION CRITERIA

Absolute contraindications include: active infection, sepsis, and osteomyelitis.

Relative contraindications include:

- Uncooperative patient or patient with neurologic disorders who are incapable of following directions
- Osteoporosis
- Metabolic disorders which may impair bone formation
- Osteomalacia
- Distant foci of infections which may spread to the implant site
- Rapid joint destruction, marked bone loss or bone resorption apparent on roentgenogram
- Vascular insufficiency, muscular atrophy, or neuromuscular disease.

Additionally, all vulnerable subjects (minors, limited or non-readers; adult subjects who cannot consent for themselves; and, pregnant women) will be excluded from participation in this study.

3.3. SUBJECT WITHDRAWAL

It is recognized that the subject's participation in this trial is entirely voluntary, and that she/he may refuse to participate and may withdraw from participation at any time without jeopardy to any future medical care. It is also recognized that the investigator, at his/her discretion, may withdraw a subject from this study based upon his/her professional judgment. In event of subject withdrawal, applicable local procedures should be followed.

If a patient is withdrawn or rescinds their consent, a "Lost to Follow-up" case report form should be completed detailing the reason for the patient withdrawal. If the patient has not yet reached the primary endpoint, the study sponsor, Biomet, should be contacted to discuss whether or not the patient should be replaced. The site should also notify their IRB or Ethical Committee if applicable. If a patient is withdrawn from the study by the investigator, the patient should be notified of their removal by a letter from the site.

It is required that patients return within the defined follow-up period to complete all study assessment forms and radiographs. Patients that miss or will not return for follow-up are not considered "protocol deviations" or "lost to follow-up."

4. PROTOCOL DEVIATION MANAGEMENT AND REPORTING

Any deviation from the protocol should be documented on the "Protocol Deviation" case report form. Protocol Deviations should be reported to Biomet within ten days of knowledge of the reported event.

5. ADVERSE EVENT MANAGEMENT AND REPORTING

Any adverse event, according to definitions section 18, should be documented on the "Complication" case report form. A record of all adverse events, including details of the nature, onset, duration, severity, relationship to the device, relationship to the operative procedure and outcome, will be made on the relevant section(s) of the subject's CRF.

Complications should be reported to Biomet within ten days of knowledge of the reported event. If the event is considered an unanticipated adverse event, it should be reported to Biomet within 5 days of knowledge of the reported event.

6. IMPLANT RETRIEVAL AND ANALYSIS OF REMOVED IMPLANTS

Should any implant failures occur, please contact the Study Manager to coordinate the implant retrieval process. The ability to analyze all removed implants will be important to the outcomes and analysis of this study.

The following requirements should be made for packaging and sending implants for analysis;

- When handling, packaging and shipping the retrieved components, the hospital or clinic should avoid putting all the components in the same container without separating packaging. Ideally, each retrieved component should be individually wrapped and stored in its own container, then placed in a larger container with all the other retrieved components. This will prevent further damage to the explants, which can be difficult to distinguish from in situ damage.
- Shortly after removal, a detailed record of any damage caused to the components during extraction should be performed, especially in the presence of the operating surgeon. Adequate 'macro' photography can also be helpful. This information should be labeled

adequately and should accompany the explanted components. This will make it easy to differentiate between damaged regions caused in vivo and those caused during extraction.

- It is imperative to label the retrieved parts adequately for future identification.
- The retrieved components should be marked in a non-destructive way to indicate their orientation in situ before removal. If the implants are not marked before extraction or immediately after, then the true location of the wear scars can only be guessed. This can be avoided easily by marking the superior rim of the cup and the base of the femoral head.
- Sterilization of metal on metal components should be performed utilizing cold techniques. Do NOT sterilize metal on metal components by steam autoclave as this may alter features or surfaces which may be essential in determining failure modes.
    - In situations where the device has already been sterilized by steam autoclave prior to coming into Biomet's control, the investigator may still perform the retrieval analysis taking into consideration the potential confounding factors introduced by the steam autoclave procedure.
    - Where residual tissues are retained on or within the components, fixation by 10% buffered formalin and/or ethanol and/or other fixative will be included in the sterilization procedure.

7. SAMPLE SIZE JUSTIFICATION

Observational study with 200 Arcos modular femoral revision cases (100 with Bonemaster and 100 without Bonemaster) to assess survivorship and revision rate up to 5 years.

The sample size for this study is based on anticipated sales (1000 during the first year of commercialization) and expected lost-to-follow-up over the first 5 years, with the goal of having a reasonable number of cases for evaluating device survival over the first 5 years of follow up.

An article by Frederick Dorey, et.al. (Journal of Arthroplasty, 1(1):63-69, 1986) recommends having greater than 20 subjects in follow-up when estimating survivorship. Lost to follow-up for this study is estimated to be 10% per year. Thus, if 100 patients of the Arcos with Bonemaster HA and 100 cases of Arcos without Bonemaster are enrolled, there will be 59 patients at 5 years of follow-up for each variant.

Start with 100 cases and assume a loss of 10% per year

- 1 year: 100-10 = 90
- 2 year: 90-9= 81
- 3 year: 81-8=73
- 4 year: 73-7= 66
- 5 year: 66-7 = 59

59 cases is a reasonable number for evaluating survivorship at 5 years per Dorey, et. al.

8. CONTROL OF INVESTIGATIONAL DEVICE

Not applicable as this is a post-market study

9. DATA COLLECTION, HANDLING AND RETENTION

9.1. SOURCE DOCUMENT REQUIREMENTS

Source documentation for this study will be maintained to document the treatment and study course of a subject and to substantiate the integrity of the trial data. Source documentation may include, but not be limited to, worksheets, hospital and/or clinic or office records documenting subject visits including study and other treatments or procedures, medical history and physical examination information, laboratory and special assessments results, pharmacy records, device accountability records, and medical consultations (as applicable).

Before the study starts, a record of the source document for each endpoint will be recorded and kept at the site and Biomet. If for any reason this source document changes, the record will need updated, and communicated to Biomet.

9.2. CASE REPORT FORMS

Data for this clinical trial will be collected and documented on the subject Case Report Forms (CRFs) provided which may be in paper form or in an electronic form. Authorized study site personnel will complete CRFs only. CRFs must be reviewed for completeness and accuracy, and signed by the Investigator or his/her designees.

Since there is a potential for errors, inaccuracies, and misinterpretation in transcribing data onto the CRFs, the following documents must be available at all times for inspection and comparison to the CRFs by the study monitor when appropriate:

- data query forms
- originals and photocopies/certified copies of all relevant records and reports
- copies of test results

Sample CRFs to be used with this clinical trial are provided in Appendix 2.

9.3. ELECTRONIC DATA ENTRY

When using electronic trial data handling and/or remote electronic trial data systems, the sponsor should:

- Ensure and document that the electronic data processing system(s) conforms to the sponsor's established requirements for completeness, accuracy, reliability, and consistent intended performance (i.e. validation).
- Maintain SOPs for using these systems.
- Ensure that the systems are designed to permit data changes in such a way that the data changes are documented and that there is no deletion of entered data (i.e. maintain an audit trail, data trail, edit trail).
- Maintain a security system that prevents unauthorized access to the data.
- Maintain a list of the individuals who are authorized to make data changes (see 4.1.5 and 4.9.3).
- Maintain adequate backup of the data.
- Safeguard the blinding, if any (e.g. maintain the blinding during data entry and processing).

9.4 STUDY DOCUMENT RETENTION

Study documents should be retained for a season after the study is complete as required by local, state, national, or international health authorities.

10. DATA REPORTING

The sponsor will present an annual report to the investigators that will include a summary of the clinical data. The report will contain the results of the Harris Hip at each time point. Also, patient follow-up will be analyzed throughout the data collection according to the following definition and equations:

Lost To Follow-Up:
1. Death
2. Revision
3. Consent Rescinded

$$\text{Percentage Follow-up} = \frac{\text{\# Patients with Follow-Up}}{\text{(Theoretically due – Lost To Follow-Up)}} \times 100$$

$$\text{Percentage Accounted for} = \frac{\text{(\# with Follow-Up + Lost To Follow-Up)}}{\text{Theoretically due}} \times 100$$

At the end of the data collection, a final report will be compiled that will summarize all data collected throughout the data collection, complications throughout the course of the data collection, and general findings.

11. RISK ANALYSIS

Please consult the package insert for the most up to date risk analysis for the product. The following warnings, precautions, and possible adverse effects associated with Total Hip Arthroplasty are as stated in the package insert for this device (cleared in 510K K100469).

A. Warnings

Improper selection, placement, positioning, alignment and fixation of the implant components may result in unusual stress conditions which may lead to subsequent reduction in the service life of the prosthetic components. Malalignment of the components or inaccurate implantation can lead to excessive wear and/or failure of the implant or procedure. Inadequate preclosure cleaning (removal of surgical debris) can lead to excessive wear. Improper preoperative or intraoperative implant handling or damage (scratches, dents, etc.) can lead to crevice corrosion, fretting, fatigue

fracture and/or excessive wear. Use clean gloves when handling implants. Do not modify implants. The surgeon is to be thoroughly familiar with the implants and instruments, prior to performing surgery.

1. Use Biomet® femoral and modular head component with appropriate matching “Type I Taper”.
2. Firmly seat modular components to prevent dissociation. Thoroughly clean and dry taper prior to attachment of the modular component to avoid crevice corrosion and improper seating.
3. Tight fixation of all non-cemented components at the time of surgery is critical to the success of the procedure. Each component must properly press fit into the host bone which necessitates precise operative technique and the use of specified instruments. Bone stock of adequate quality must be present and appraised at the time of surgery.
4. Complete preclosure cleaning and removal of metallic debris and other surgical debris at the implant site is critical to minimize wear of the implant articular surfaces.
5. Distal cross-screws are intended to provide temporary (<6 months) rotational stability only. Cross-screws are not intended to carry any axial load.

Biomet® joint replacement prostheses provide the surgeon with a means of reducing pain and restoring function for many patients. While these devices are generally successful in attaining these goals, they cannot be expected to withstand the activity levels and loads of normal, healthy bone and joint tissue.

Accepted practices in postoperative care are important. Failure of the patient to follow postoperative care instructions involving rehabilitation can compromise the success of the procedure. The patient is to be advised of the limitation of the reconstruction and the need for protection of the implants from full load bearing until adequate fixation and healing have occurred. Excessive, unusual and/or awkward movement and/or activity, trauma, weight gain, and obesity have been implicated with premature failure of the implant by loosening, fracture, dislocation, subluxation and/or wear. It has been noted that this may be particularly true where smaller sized stems are involved. Loosening of the implants can result in increased production of wear particles, as well as accelerate damage to bone, making successful revision surgery more

difficult. The patient is to be made aware and warned of general surgical risks, possible adverse effects as listed, and to follow the instructions of the treating physician, including follow-up visits.

Patient selection factors to be considered include: 1) need to obtain pain relief and improve function, 2) ability and willingness of the patient to follow instructions, including control of weight and activity level, 3) a good nutritional state of the patient, and 4) the patient must have reached full skeletal maturity.

Patient smoking may result in delayed healing, non-healing and/or compromised stability in or around the placement site.

B. Precautions

Specialized instruments are designed for Biomet® joint replacement systems to aid in the accurate implantation of the prosthetic components. The use of instruments or implant components from other systems can result in inaccurate fit, sizing, excessive wear and device failure. Intraoperative fracture or breaking of instruments has been reported. Surgical instruments are subject to wear with normal usage. Instruments, which have experienced extensive use or excessive force, are susceptible to fracture. Surgical instruments should only be used for their intended purpose. Biomet recommends that all instruments be regularly inspected for wear and disfigurement.

Do not reuse implants. While an implant may appear undamaged, previous stress may have created imperfections that would reduce the service life of the implant. Do not treat patients with implants that have been, even momentarily, placed in a different patient.

C. Possible Adverse Effects

1. Material sensitivity reactions. Implantation of foreign material in tissues can result in histological reactions involving various sizes of macrophages and fibroblasts. The clinical significance of this effect is uncertain, as similar changes may occur as a precursor to or during the healing process. Particulate wear debris and discoloration from metallic and polyethylene components of joint implants may be present in adjacent tissue or fluid. It has been reported that wear debris may initiate a cellular response resulting in osteolysis or osteolysis may be a result of loosening of the implant. Further, there has been a report

regarding an association between articulating surfaces of: 1) CoCrMo alloy on CoCrMo alloy, 2) CoCrMo alloy on polyethylene, and 3) Titanium alloy on polyethylene in hip replacements and increased genotoxicity. This report, however, did not assess either the clinical relevance of the data or make any definite conclusions as to which metal ions or interactions between metal ions or particulate metals might be responsible for the observed data. The report further cautioned that an association does not necessarily mean a causal relationship, and that any potentially increased risk associated with metal ions needs to be balanced against the benefits resulting from hip replacement.

2. Early or late postoperative infection and allergic reaction.
3. Intraoperative bone perforation or fracture may occur, particularly in the presence of poor bone stock caused by osteoporosis, bone defects from previous surgery, bone resorption, or while inserting the device.
4. Loosening or migration of the implants can occur due to loss of fixation, trauma, malalignment, malposition, bone resorption, or excessive unusual and/or awkward movement and/or activity.
5. Periarticular calcification or ossification, with or without impediment of joint mobility.
6. Inadequate range of motion due to improper selection or positioning of components.
7. Undesirable shortening of limb.
8. Dislocation and subluxation due to inadequate fixation, malalignment, malposition, excessive, unusual and/or awkward movement and/or activity, trauma, weight gain, or obesity. Muscle and fibrous tissue laxity can also contribute to these conditions.
9. Fatigue fracture of component can occur as a result of loss of fixation, strenuous activity, malalignment, trauma, non-union, or excessive weight.
10. Fretting and crevice corrosion can occur at interfaces between components.
11. Fracture of the cross-screws.
12. Wear and/or deformation of articulating surfaces.
13. Trochanteric avulsion or non-union as a result of excess muscular tension, early weight bearing, or inadequate reattachment.
14. Problems of the knee or ankle of the affected limb or contralateral limb aggravated by leg length discrepancy, too much femoral medialization or muscle deficiencies.
15. Postoperative bone fracture and pain.

12. MONITORING PLAN

Biomet, as the sponsor of this study, may monitor the data collection to ensure that the investigation is being conducted consistent with the protocol. The following describes the monitoring activities, which may take place during the course of the study.

12.1 FREQUENCY

Pre-Investigational Visit/Conference:

Prior to initiation of the study, the study manager will provide the Investigator with all the necessary information to enable him to carry out his responsibilities. This prepares the site with an in-depth training on the protocol, case report forms, and data collection process for the length of the study. The study manager will also train the site on using the Biomet Joint Assist database.

Monitoring of the Data

Europe: Monitoring of the data will occur at least annually, and as often as monthly. Times when this may be appropriate include:

- Monthly Invoicing
- Quarterly Review
- Annual Reports
- While performing data analysis for marketing material or publication.

United States: Since data is entered electronically, monitoring of the study will be done centrally, and no onsite monitoring will occur.

12.2 SAMPLING PLAN

All data will be monitored for completeness and accuracy on at least an annual basis.

12.3 MONITORING TASKS

Biomet will continually monitor the progress of the clinical trial. These activities include:

- Tracking of patient enrollment
- Review of all electronic patient data forms received by Biomet for completeness
- Tracking of patients to ensure follow-ups are being completed at appropriate intervals

- Review of all adverse reactions
- Maintaining open communication with all investigational sites in order to ensure the quality of the clinical trial.
- In-House Audits as needed

Upon completion of any type of monitoring, the site is responsible for resolving all discrepancies found in a timely manner. These will be sent to the site with an audit report by the study manager. All discrepancies found within the Joint Assist database will be queried and sent directly to the site. Delays in resolving queries are to be avoided at all costs; this provides the study with the most accurate data, prevents delay in reporting procedures & publication, and safeguards in the event of an audit by the relative regulatory authority in the region.

12.4 STUDY CLOSE-OUT

When a site has completed their data collection, a visit may be necessary by a Biomet monitor to ensure all data has been obtained. Data will be reviewed for completeness, and monitored to ensure that all discrepancies have been resolved.

13. LABELING

The devices and products will be used in accordance with their instructions for use and/or approved labeling. The package insert for the device(s) in this study is included in the Investigator Binder.

14. ETHICAL AND REGULATORY REQUIREMENTS*

14.1. CODE OF CONDUCT

The Investigator will ensure that the clinical study is conducted in accordance with

1. Protocol
2. Regulatory and IRB/EC requirements
    a. In the United States: Premarket Approval P010014

   b. CE Marked product: must conform to the essential requirements of EC Directive 93/42/EEC; in accordance with 13485:2003.

3. ISO 14155, GCP

14.2. REGULATORY APPROVAL (i.e. FDA, MHRA, MHLW/PMDA)

NOT APPLICABLE: The devices used in this study are post-market devices already approved for use in the United States and Europe.

14.3. INSTITUTIONAL REVIEW BOARDS (if applicable by the local country)

The Investigator must obtain appropriate Independent Ethics Committee (IEC) approval before the study can be initiated. A copy of the written approval from the IEC and a copy of the approved informed consent form should be sent to the Sponsor. A list of the IEC members (including their Institution affiliations, gender makeup, and occupations); or a statement from the IEC specifying that the membership comply with applicable regulations is to be provided to the sponsor. This must be done on an annual basis and copies sent to the sponsor as long as the study is open at the site.

Any changes to the protocol must be discussed and approved by the Sponsor in writing unless the change is made to assure the safety of the subject. In the non-emergent setting, after agreement on the changes has been reached, an amendment to the protocol will be provided by the Sponsor for submission to the IEC for review and approval prior to initiation of the change. Any change made emergently must be documented in the subject's medical record and reported to the Sponsor within the time period required by local SOPs and applicable regulations.

The Investigator must immediately forward to the IEC any written safety reports or updates from the Sponsor.

The Investigator must keep the IEC informed of the progress of the study as required by the IEC but at least annually.

14.4. INFORMED CONSENT

Subjects (or the subject's legally authorized representative) will be provided with an informed consent and patient information sheet in order to give ample opportunity to review the consent and ask questions. Since all patients have already been treated with revision surgery, the signed informed consent will be obtained at patients next prospective follow-up visit, before any prospective study procedures begin. If the subject agrees to participate in the study, the subject/representative must sign the informed consent form. The witness and the Investigator must also sign the informed consent form. A copy of the informed consent form should be given to the subject/representative. All subjects who meet all of the entry criteria will be considered for inclusion in this trial. Any subject meeting any of the exclusion criteria will be excluded from the trial.

The informed consent form must be approved by the institution's IEC, or other locally required reviewing body.

Subjects will be informed of new information learned during the study, which may affect the subject's decision to continue participation in the study.

An Informed Consent Log will be completed to document the existence of the signed informed consent form. The log will contain: Subject ID, date informed consent form signed, and the version signed. The monitor will initial and date the log once the executed informed consent form has been reviewed. Signed informed consent forms (or copies) are to be maintained in the study file and must be available for verification by monitors or inspectors.

14.5. SUBJECT CONFIDENTIALITY

The case report forms do not include any patient identifying information. Therefore, once the data is entered in the online database a patient can no longer be identified.

Once the site enters a patient into Joint Assist, the database will assign the patients an ID number. It is the responsibility of the investigator to maintain a list of patient identification and Joint Assist ID numbers throughout the course of the study. By assigning patients a unique ID number, their identity is protected in Joint Assist, the online database. The database is restricted, allowing a doctor to only view and enter data from his own patients. User authentication is required to view research data. The data is transmitted to a centralized database through a

secured (SSL) channel on the Internet. Data in transit is in 128-bit encryption. The access to the centralized database is limited to those who are responsible for maintaining the database.

15. INSURANCE AND INDEMNIFICATION

This arrangement will be negotiated by site in their investigator agreement.

16. STUDY DEFINITIONS

Not Applicable

17. REFERENCES

Not applicable

18. APPENDICS

Appendix 1 Informed Consent Draft for Use in Submission to Ethical Committee or IRB

Appendix 2 Case Report Forms

Appendix 3 PMA Approval / CE Mark Info / Package Insert (as applicable by region)

Appendix 4 Surgical Technique & Product Brochures

Not applicable

SIGNATURE PAGE

Protocol authorized by (Project Leader):

Version	Name & Role	Date	Signature
Version 1, 2, 3, 4	Jason Critchlow – Clinical Research Associate	April 2, 2013	Previously Signed
Version 5	Megan King, Manager, Clinical Operations	January 13, 2014	Previously signed
Version 6:<br><br>Including retrospective data collection	Lisa To, project leader	March 18, 2015	
	Gregoire Edorh, Global hip & cement clinical strategy	March 18, 2015	
